# New Imaging Signatures of Cardiac Alterations in Ischaemic Heart Disease and Cerebrovascular Disease Using CMR Radiomics

**DOI:** 10.3389/fcvm.2021.716577

**Published:** 2021-09-23

**Authors:** Elisa Rauseo, Cristian Izquierdo Morcillo, Zahra Raisi-Estabragh, Polyxeni Gkontra, Nay Aung, Karim Lekadir, Steffen E. Petersen

**Affiliations:** ^1^William Harvey Research Institute, National Institute for Health Research Barts Biomedical Research Centre, Queen Mary University of London, Charterhouse Square, London, United Kingdom; ^2^Barts Heart Centre, St Bartholomew's Hospital, Barts Health National Health Service Trust, London, United Kingdom; ^3^Departament de Matematiques i Informatica, Universitat de Barcelona, Artificial Intelligence in Medicine Lab, Barcelona, Spain; ^4^Health Data Research UK, London, United Kingdom; ^5^Alan Turing Institute, London, United Kingdom

**Keywords:** cardiovascular magnetic resonance, radiomics, ischaemic heart disease, myocardial infarction, cerebrovascular disease, stroke, brain-heart axis

## Abstract

**Background:** Ischaemic heart disease (IHD) and cerebrovascular disease are two closely inter-related clinical entities. Cardiovascular magnetic resonance (CMR) radiomics may capture subtle cardiac changes associated with these two diseases providing new insights into the brain-heart interactions.

**Objective:** To define the CMR radiomics signatures for IHD and cerebrovascular disease and study their incremental value for disease discrimination over conventional CMR indices.

**Methods:** We analysed CMR images of UK Biobank's subjects with pre-existing IHD, ischaemic cerebrovascular disease, myocardial infarction (MI), and ischaemic stroke (IS) (*n* = 779, 267, 525, and 107, respectively). Each disease group was compared with an equal number of healthy controls. We extracted 446 shape, first-order, and texture radiomics features from three regions of interest (right ventricle, left ventricle, and left ventricular myocardium) in end-diastole and end-systole defined from segmentation of short-axis cine images. Systematic feature selection combined with machine learning (ML) algorithms (support vector machine and random forest) and 10-fold cross-validation tests were used to build the radiomics signature for each condition. We compared the discriminatory power achieved by the radiomics signature with conventional indices for each disease group, using the area under the curve (AUC), receiver operating characteristic (ROC) analysis, and paired *t*-test for statistical significance. A third model combining both radiomics and conventional indices was also evaluated.

**Results:** In all the study groups, radiomics signatures provided a significantly better disease discrimination than conventional indices, as suggested by AUC (IHD:0.82 vs. 0.75; cerebrovascular disease: 0.79 vs. 0.77; MI: 0.87 vs. 0.79, and IS: 0.81 vs. 0.72). Similar results were observed with the combined models. In IHD and MI, LV shape radiomics were dominant. However, in IS and cerebrovascular disease, the combination of shape and intensity-based features improved the disease discrimination. A notable overlap of the radiomics signatures of IHD and cerebrovascular disease was also found.

**Conclusions:** This study demonstrates the potential value of CMR radiomics over conventional indices in detecting subtle cardiac changes associated with chronic ischaemic processes involving the brain and heart, even in the presence of more heterogeneous clinical pictures. Radiomics analysis might also improve our understanding of the complex mechanisms behind the brain-heart interactions during ischaemia.

## Introduction

Ischaemic heart disease (IHD) and ischaemic cerebrovascular diseases are the leading causes of death and disability worldwide (1). Although each entity has its own particularities, both share common pathophysiological mechanisms mostly linked to atherosclerosis and atherothrombosis. Patients with these ischaemic conditions share similar clinical profiles, and their co-existence is common (2).

Multiple interactions occur among these two disease entities, which are not fully explained by shared vascular risk factors (3, 4). Several mechanisms of brain-heart interaction have been hypothesised, indicating complex bidirectional pathways between the two diseases. In this scenario, cardiac diseases may be the underlying cause of cerebrovascular events, while cerebral ischaemia, in turn, may be associated with disturbances in heart function (5).

Previous studies have focused on the possible brain areas involved in the crosstalk between the two systems. They suggest that cerebral ischaemia might trigger different pathways of dysautonomia and increased inflammation, potentially resulting in cardiac dysfunction (6–8). The main long-term cardiac abnormalities described on conventional images include left ventricle (LV) systolic and diastolic dysfunction, which can both lead to cardiac remodelling (9, 10). However, whether there are more subtle cardiac changes after ischaemic events that might contribute to cardiac remodelling has not yet been thoroughly investigated.

Cardiovascular magnetic resonance (CMR) radiomics analysis may provide new insights into the quantitative analysis of cardiac imaging by extracting a large number of computational quantitative metrics, including shape and texture features, which may capture a wide variety of phenotypic traits. Several studies have already demonstrated their incremental role over conventional imaging indices in identifying subtle cardiac alterations due to specific clinical conditions (11).

Studying the morphological and myocardial tissue changes associated with ischaemic heart and brain diseases using CMR radiomics analysis may provide new insight into the relationship between these two systems and their underlying mechanisms. These additional imaging markers may also improve the early diagnosis of ischaemic events and help clinicians identify those most at risk who require more aggressive preventive strategies.

In this paper, we used CMR radiomics analysis to study whether there were specific cardiac abnormalities in individuals who had previous ischaemic cerebrovascular events and cardiac events (IHD). We used machine learning (ML) methods to identify the most defining radiomics features for each condition, called “radiomics signature.” Finally, we studied whether the radiomics-based models provided incremental information to conventional approaches. To our knowledge, no previous studies have used this approach to investigate the brain-heart interactions after ischaemic processes on a large cohort of participants as it is the United Kingdom Biobank (UKB).

## Methods

### The UK Biobank Dataset

In this work we used clinical and imaging data from UKB, a prospective cohort study which has been following the health and well-being of half a million participants aged 40–69 years-old recruited from across the UK between 2006 and 2010 (12). Its goal is to improve the prevention, diagnosis, and treatment of a wide range of severe and life-threatening illnesses. These resources are made available to health researchers through an application process. Information on participants' health, lifestyle, hearing and cognitive function, family history as well as physical measurements, biological samples, and genome were collected at recruitment (13). Some baseline measurements were updated in subsequent visits. UKB is linked to a range of electronic health records (cancer, death, hospital episodes, and general practise) which provide information related to participants' health outcomes. UKB also provides algorithmically defined health outcomes, a classification of selected health-related events, obtained through the algorithmic combinations of baseline information along with linked data from hospital admissions and death registries. This classification allows for accurate identification of diseases and their sub-sets. Since 2015, over 48,000 UKB participants (April 2021) underwent CMR. The extensive amount of data available from each participant, make UKB a good resource to address brain-heart interactions.

### Defining the Study Populations

Among all the participants from UKB who completed the CMR imaging protocol and passed the quality control check, we identified subjects with a previous diagnosis of IHD and those with cerebrovascular disease using a combination of selected ICD-10 and ICD-9 codes for diagnosis. Participants who had both conditions at the time of the CMR study were excluded from the study. The full list of ICD codes for each study group is provided in Supplementary Table 1.

We initially identified 1,516 subjects with IHD, including angina, previous myocardial infarction (MI), or any manifestation of IHD not resulting in MI.

The ischaemic cerebrovascular disease subset, instead, included previous ischaemic stroke (IS) or transient ischaemic attack (TIA), as both clinical manifestations have a significant prognostic value (14, 15) (*n* = 267).

Participants with MI (*n* = 525) and IS (*n* = 107) only were also considered separately as the subsets with evidence of cardiac and cerebral organ damage, respectively. Definitions for MI were consistent with those used by the UKB outcome adjudication group (16). The identification of IS and TIA derived from the ICD codes was in line with what suggested in a Systematic Review from the UK Biobank Stroke Outcomes Group to identify only the cases representative of the disease subtype studied with adequate sensitivity and positive predictive value (17).

We chose as a comparator group the healthy Caucasian “reference” cohort previously identified by Petersen et al. (18) to establish the reference ranges for cardiac structure and function in CMR (*n* = 779). We selected this cohort because it was well-validated, and the rule-out criteria used to define the “healthy” status were robust. Subjects were thus considered healthy if they did not have any known cardiovascular diseases, traditional risk factors, or other systemic conditions that could have impacted the heart at the time of CMR study. The complete list of exclusion criteria used to define the selected healthy cohort has been described elsewhere (18).

Each disease group was then compared with an equal number of randomly selected healthy controls to avoid class imbalance problems, potentially affecting the ML models. Therefore, this process produced four distinct control groups, each per disease group.

Since there were fewer healthy participants than those with IHD (*n* = 779 vs. 1,516, respectively), to obtain balanced groups, we finally selected only 779 IHD subjects to match the numbers of healthy comparators.

Therefore, at the end of the selection process, we had four disease subsets each one to be compared with an equal number of healthy controls: IHD (*n* = 779), cerebrovascular disease (*n* = 267), MI (*n* = 525), and IS (*n* = 107).

**Figure 1:**
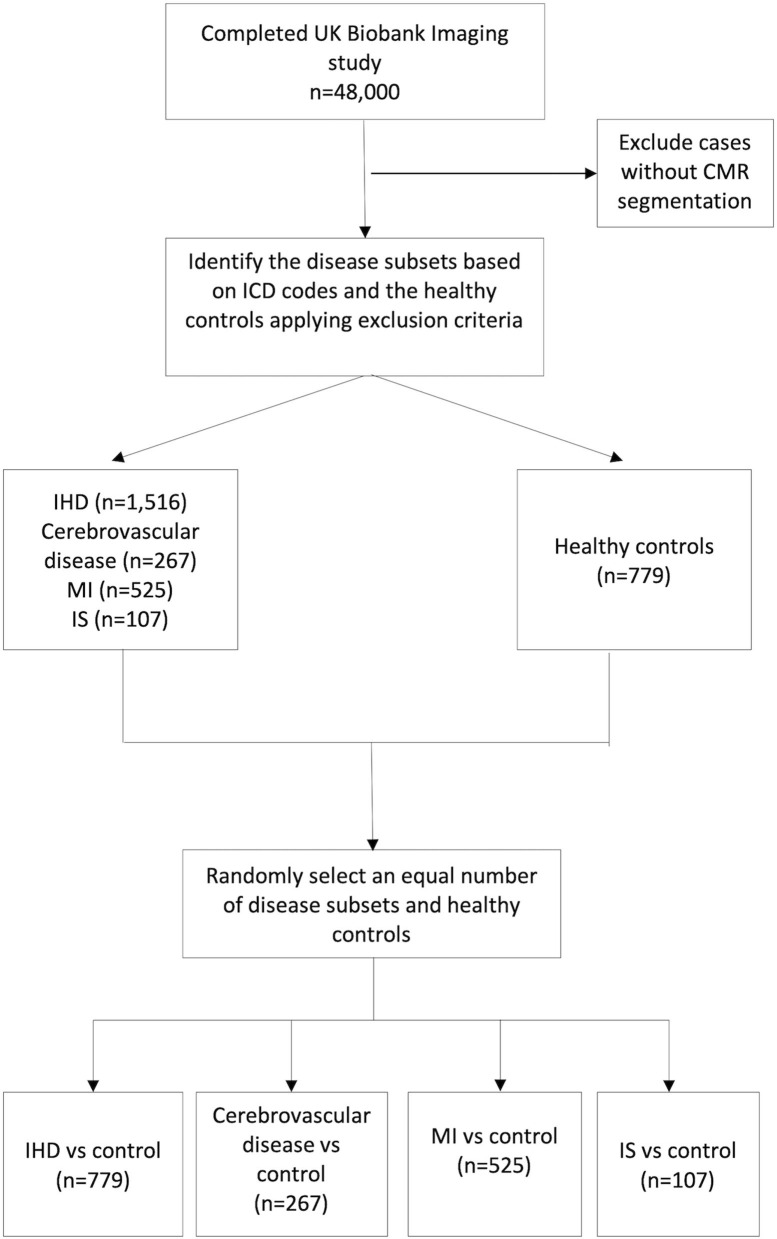
Study cohorts selection process. IHD, Ischaemic heart disease; MI, myocardial infarction; IS, ischaemic stroke.

The overall participants' selection process is shown in Figure 1.

We thus conducted two experiments using different study groups for the analysis.

In one experiment, we focused only on MI and IS subsets to see whether ischaemia resulting in organ damage was associated with cardiac changes detected on radiomics analysis.

In a second experiment, we analysed the radiomics features of the IHD and cerebrovascular disease subsets. Our goal was to verify whether it was possible to capture common cardiac alterations associated with more clinically heterogeneous ischaemic conditions.

### CMR Imaging Protocol and Segmentation

The full CMR protocol in UKB has been described in details elsewhere (19). In brief, the CMR images were acquired with 1.5 Tesla scanner (MAGNETOM Aera, Syngo Platform VD13A, Siemens Healthcare, Erlangen, Germany) with an in-plane resolution of 1.8 × 1.8 mmsq, a slice thickness of 8.0 mm and a slice gap of 2 mm. Cardiac function was assessed on a combination of long axis (LAX) cines (horizontal long-axis—HLA, vertical long-axis—VLA, and left ventricular outflow tract—LVOT cines, both sagittal and coronal) and a complete short-axis (SAX) stack covering the left ventricle (LV) and right ventricle (RV). All cine images were acquired at one slice per breath-hold.

CMR image segmentation was performed manually in the initial ~5,000 studies by two image-analysis core laboratories as previously described (18). The manually segmented CMR studies were visually quality checked at the time of image annotation; 153 studies were excluded due to poor image quality.

Using this expert-annotated dataset a U-net based fully convolutional neural network was trained to annotate the remaining UKB CMR studies (*N* = 16,186) (20). Among the automatically segmented CMR studies, we reviewed 1,569 studies with outlying measurements. Almost all cases that had to be removed were due to the quality issues related to incomplete LV coverage or image artefacts from mis-triggering or poor breath-hold. Studies with segmentation failure due to image quality issues were excluded from downstream analysis. Overall, automatic segmentation produced robust LV contours as indicated by the Dice scores (0.88–0.94). Details of reproducibility performance of the automated algorithm are available in dedicated publications (18, 21). From the SAX cine CMR images, the LV and RV endocardial contours and LV epicardial contours were traced in both end-diastole (ED) and end-systole (ES) using the automated tool. From the automated segmentation, we defined three regions of interest (ROI): LV blood pool (LV), LV myocardium (MYO), and RV blood pool (RV).

### Conventional CMR Indices

Conventional CMR indices of cardiac structure and function were also assessed and compared to the added value of CMR radiomics. In particular, we automatically calculated: LV end-diastolic volume (LVEDV), LV end-systolic volume (LVESV), RV end-diastolic volume (RVEDV), RV end-systolic volume (RVESV), LV stroke volume (LVSV), RV stroke volume (RVSV), LV ejection fraction (LVEF), RV ejection fraction (RVEF), LV mass (LVM). The CMR parameters were subsequently indexed to the subject's body surface area (BSA) and used for further analysis.

### CMR Radiomics Analysis

From the three segmented ROIs in ED and ES we extracted radiomics shape and signal intensity-based features using the open-source packages Pyradiomics version 2.2.0 (22).

Radiomics shape features quantify size and shape of the segmented ROIs and have the potential to capture subtle geometrical and morphological alterations of the cardiac structures beyond those described by conventional CMR indices. Shape features include conventional indices, such as cavity volume, and more advanced geometric quantifiers, such as sphericity, compactness, and elongation.

Signal intensity-based radiomics features are grouped into two categories: first-order and texture features. These features describe the global distribution and pattern of voxel signal intensity (SI) and may potentially capture changes in cardiac tissue induced by disease processes.

In particular, first-order radiomics describe the global SI distribution within the ROIs by plotting a histogram and without focusing on their spatial relationships. The global SIs distribution is described using simple quantifiers, such as mean, median, and standard deviation, and more advanced measures, such as skewness, uniformity, or entropy.

Texture features, instead, describe the SIs patterns within the segmented ROIs considering the relationship of the voxel SIs to each other. Various mathematical processes are used to quantify the complexity, coarseness and repeatability of the SI matrix (23, 24). Texture features may reflect the myocardial tissue characteristics related to a specific disease.

It should be noted that radiomics are sensitive to intensity variations related to the image acquisition process. Therefore, to account for such variations and increase their repeatability, prior to feature extraction, intensity normalisation within the heart region was performed by means of histogram matching using as reference one of the CMR from the UKB (25).

### Radiomics Feature Selection

Radiomics shape and signal intensity-based features were extracted from the three ROIs (LV, MYO, and RV) in ED and ES. RV first-order and RV texture radiomics features were not considered for the analysis as deemed not clinically relevant. This resulted in a total of 446 radiomics features to be included in the study.

From this subset of features a highly correlated removal was applied as many of them were not independent and were expected to encapsulate redundant information. Towards this aim, we used Pearson correlation coefficient higher than 0.9, and we retained only one of the correlated features, resulting in 261, 267, 267, and 265, on average per fold, for IHD, MI, cerebrovascular disease and IS, respectively.

After this data pre-processing, a ML grid search pipeline was performed, including a feature selection step. KBest algorithm based on mutual information was selected among different feature selection (FS) techniques due to its robustness and performance efficiency. Other FS techniques, such Sequential Feature Selection (SFS) were discarded due to high computational times and equal performance.

### Radiomics Signatures and ML Modelling

Our study aimed to identify radiomics signatures that best describe the cardiac structural and tissue changes occurring in ischaemic heart and brain diseases to differentiate subjects with these conditions from healthy.

For that purpose, the most informative sets of k radiomics features were selected based on the FS algorithm described in the previous section and fed to ML classifiers to discriminate between each disease vs. control group (healthy cohort). Data from the different cohorts was properly under sampled to match equally distributed cohorts for comparison.

Among different state-of-the-art ML algorithms evaluated using a simple splitting of the dataset [support vector machines (SVM), random forests (RF), XGboost, decision trees, and multilayer perceptron and naive Bayes classifier], SVM and RF performed consistently better. Therefore, we decided to use these two algorithms to build our ML models. In this paper, we describe only the results obtained with SVM and RF in a 10-fold cross validation grid search and hyperparameter tunning, and tested in a 10-fold unseen dataset (further details about the ML models building are described in the Appendix).

For clinical validation, we compared the radiomics signatures to similar ML models based on conventional CMR indices. To further test the potential incremental value of radiomics, we also evaluated the performance achieved using a combined model containing both radiomics and traditional metrics for each disease group.

Paired *t*-test was used to assess the statistical significance of the differences between radiomics and combined models vs. conventional indices. A *p* < 0.05 was considered to be statistically significant. The models' performance was assessed using the receiver operating characteristic (ROC) curve analysis and area under the ROC curve (AUC) score. Group differences were evaluated using independent *t*-tests after assessing the normal distribution of the data. Statistical analysis was performed using Python Version 3.6.4 (Python Software Foundation, Delaware USA).

## Results

### Baseline Characteristics

At the end of the selection process, the subjects with IHD, cerebrovascular disease, MI, and IS available for the analysis were 779, 267, 525, and 107, respectively.

Baseline characteristics and CMR indices for each disease group and healthy controls are shown in Table 1.

**Table 1:** Baseline characteristics and conventional CMR measurements for each disease group and the healthy controls.

**Parameter**	**IHD** **(*n* = 779)**	**Cerebrovascular disease** **(*n* = 267)**	**MI** **(*n* = 525)**	**IS** **(*n* = 107)**	**Healthy** **(*n* = 779)**
**Baseline characteristics**					
Age, years	67 ± 6^*^	68 ± 6^*^	67 ± 6^*^	67 ± 7^*^	59 ± 7
Female gender, *n* (%)	210 (27)^*^	99 (37)^*^	89 (17)^*^	37 (35)^*^	420 (54)
Body mass index (kg/m^2^)	28.1 ± 4.3^*^	27.8 ± 4.6^*^	28.2 ± 4.3^*^	28.1 ± 4.7^*^	24.4 ± 2.7
Body surface area (m^2^)	1.9 ± 0.2^*^	1.9 ± 0.2^*^	1.9 ± 0.3^*^	1.9 ± 0.2^*^	1.8 ± 0.2
Diabetes, *n* (%)	70 (9)	17 (6)	41 (8)	6 (6)	–
Hypertension, *n* (%)	343 (44)	96 (36)	255 (48)	40 (37)	–
High cholesterol, *n* (%)	452 (58)^†^	119 (44)	328 (62)^†^	42 (39)	–
Smoker, *n* (%)	62 (8)	16 (6)	52 (10)	9 (8)	–
**Conventional CMR indices**					
LVEDV index (ml/m^2^)	81.2 ± 17.1	76.1 ± 14.4^*^	86.0 ± 19.2^*^	75.9 ± 15.0^*^	81.1 ± 14.0
LVESV index (ml/m^2^)	35.01 ± 12.9^*^	31.6 ± 8.5	39.2 ± 15.7^*^	31.7 ± 8.6	33.3 ± 8.0
LVSV index (ml/m^2^)	46.2 ± 8.6^*^	44.5 ± 8.8^*^	46.8 ± 8.9	44.2 ± 9.0^*^	47.8 ± 9.1
LVM index (g/m^2^)	48.9 ± 9.5^*^	46.9 ± 9.3	51.1 ± 9.8^*^	46.3 ± 8.0	45.2 ± 9.5
RVEDV index (ml/m^2^)	82.9 ± 14.8^*^	80.2 ± 15.8^*^	84.6 ± 14.9	80.0 ± 15.2^*^	85.9 ± 16.9
RVESV index (ml/m^2^)	36.2 ± 9.2^*^	35.2 ± 9.5^*^	37.4 ± 9.0	35.0 ± 9.0^*^	38.5 ± 11.2
RVSV index (ml/m^2^)	46.6± 8.9	44.9 ± 9.7^*^	47.2 ± 9.3	44.9 ± 9.9	47.4 ± 8.5
LVEF (%)	57.7 ± 7.6^*^	58.7 ± 6.3	55.3 ± 8.4^*^	58.5 ± 6.3	59.1 ± 5.7
RVEF (%)	56.5 ± 6.5	56.2 ± 6.9	55.9 ± 6.6	56.2 ± 7.1	55.7 ± 6.2

*IHD, Ischaemic heart disease; MI, Myocardial infarction; IS, Ischaemic stroke; LVEDV, left ventricle end-diastolic volume; LVESV, left ventricle end-systolic volume; LVSV, left ventricle stroke volume; LVM, left ventricle mass; RVEDV, right ventricle end-diastolic volume; RVESV, right ventricle end-systolic volume; RVSV, right ventricle stroke volume; LVEF, left ventricle ejection fraction; RVEF, right ventricle ejection fraction. Data are presented as means ± standard deviations for continuous variable and count (%) for categorical variables*.

* *p < 0.001 when compared with healthy controls*.

† *p < 0.001 when compared with cerebrovascular disease and IS*.

The average age was similar across the disease groups (from 67 ± 6 to 68 ± 6 years) and it was significantly higher than the healthy controls (59 ± 7 years). The majority of participants were male, while the highest percentage of female subjects was observed in cerebrovascular disease and IS cohorts (37 and 35%, respectively). Furthermore, the distribution of cardiovascular risk factors (diabetes, hypertension, and smoking) was similar across the disease groups, except for hypercholesterolaemia, which percentage was significantly higher in IHD and MI.

Subjects with previous MI had significantly higher LV volumes and LVM index and lower LVEF values than the healthy control. A similar pattern was observed in the more heterogeneous cohort of IHD, where significantly lower RV volumes were also observed.

IS and cerebrovascular disease, instead, had the lowest indexed LVEDV and RV volumes among the disease subsets, while the cardiac function indices were similar to the healthy group.

### Comparing the Discriminative Performance of Radiomics Only, Conventional CMR Indices, and Combined Models

**Figure 2:**
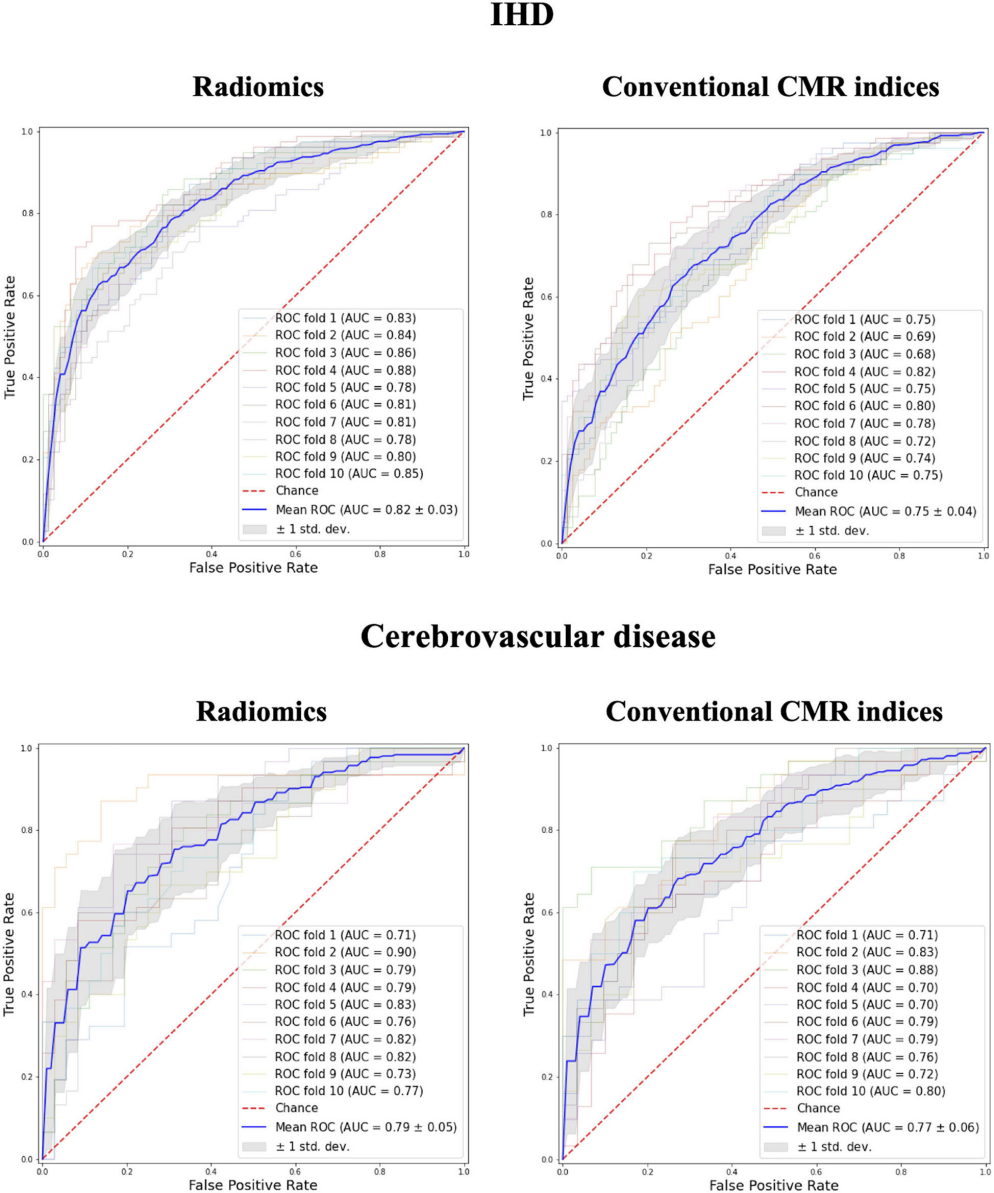
Receiver operating curves for radiomics vs. conventional CMR indices models in IHD and Cerebrovascular Disease groups. IHD, ischaemic heart disease; AUC, Area under the curve.

In comparison to conventional CMR indices, radiomics provided a better degree of discrimination between healthy and subjects with IHD (0.82 AUC for radiomics vs. 0.75 for conventional indices) and cerebrovascular disease (0.79 vs. 0.77; Figure 2).

**Figure 3:**
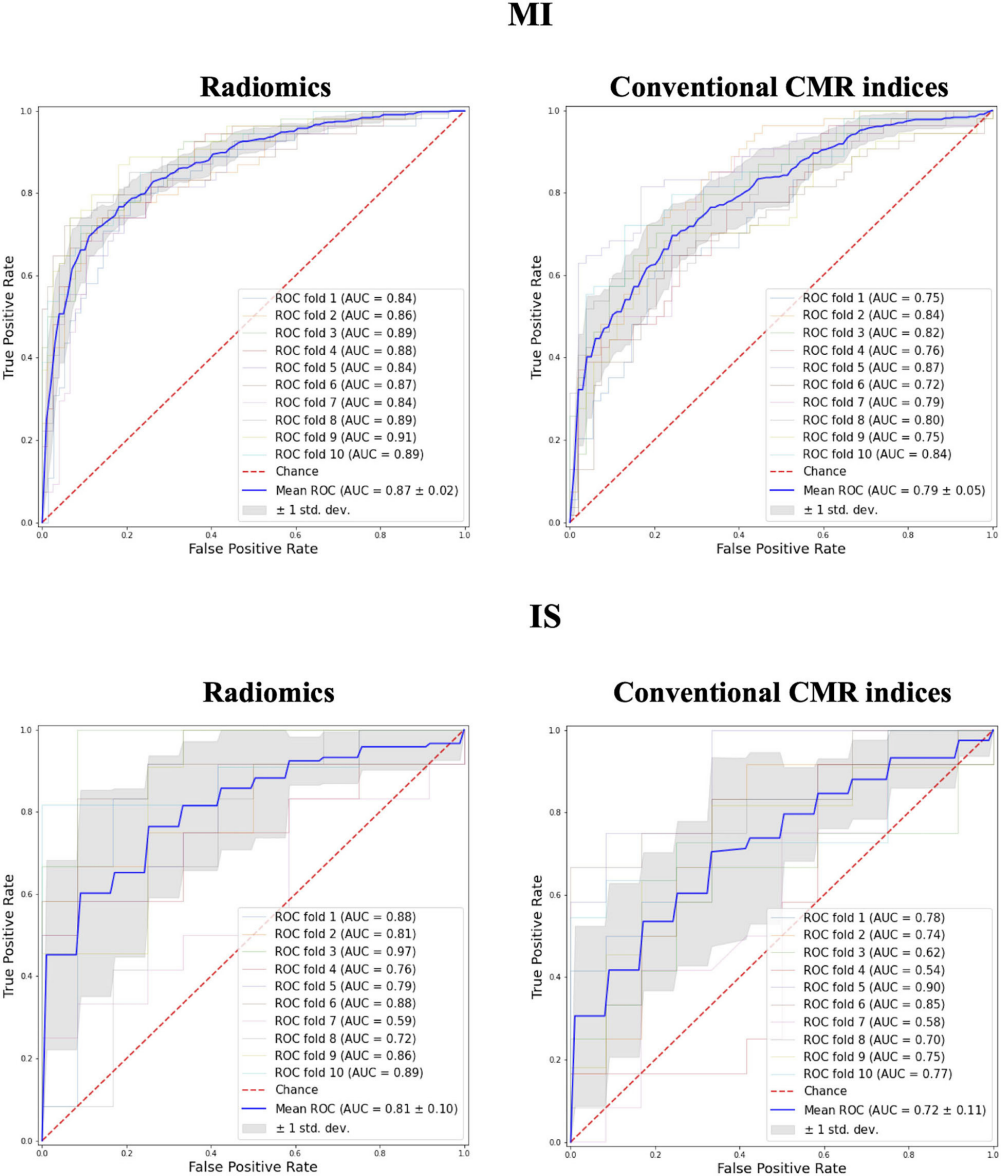
Receiver operating curves for radiomics vs. conventional CMR indices models in MI and IS groups. MI, myocardial infarction; IS, ischaemic stroke; AUC, Area under the curve.

A marginally higher degree of discrimination with radiomics model was observed in MI and IS groups (MI: 0.87 AUC for radiomics vs. 0.79 for conventional indices; IS: 0.81 AUC for radiomics vs. 0.72 for conventional indices; Figure 3).

The degree of discrimination achieved by the combined models was similar to that observed with radiomics only (Supplementary Figure 1). In particular, the best performing combined models provided significant incremental value over conventional CMR indices for all disease groups, similar to that achieved with radiomics only (Table 2).

**Table 2:** Comparing the degree of discrimination achieved using radiomics only, conventional CMR indices and both (combined model) for each disease group.

**Model**	**Radiomics**	**Conventional CMR indices**	**Combined**	***p*-value**
**IHD**				
SVM	0.82 (0.03)	0.75 (0.04)	0.83 (0.03)	<0.05
Random Forest	0.80 (0.04)	0.69 (0.05)	0.82 (0.04)	<0.05
**Cerebrovascular disease**				
SVM	0.79 (0.05)	0.77 (0.06)	0.81 (0.05)	<0.05
Random Forest	0.76 (0.05)	0.69 (0.07)	0.79 (0.02)	<0.05
**MI**				
SVM	0.87 (0.02)	0.79 (0.05)	0.86 (0.02)	<0.05
Random Forest	0.83 (0.04)	0.77 (0.03)	0.83 (0.02)	<0.05
**IS**				
SVM	0.81 (0.10)	0.72 (0.11)	0.81 (0.10)	0.08
Random Forest	0.77 (0.08)	0.67 (0.07)	0.78 (0.09)	<0.05

*The model performance is presented as mean AUC (STD). The paired t-test statistical significance is used to compare combined model vs. conventional CMR indices*.

*IHD, Ischaemic heart disease; MI, myocardial infarction; IS, ischaemic stroke; SVM, Support Vector Machines; RF, Random Forests; AUC, Area under the curve; STD, Standard deviation*.

### Identifying Radiomic Signatures for Each Disease Group

The final number of features selected for the model was 261 in IHD, 267 in MI, 267 in cerebrovascular disease, and 265 in IS, and included both shape, first-order and texture radiomics.

**Figure 4:**
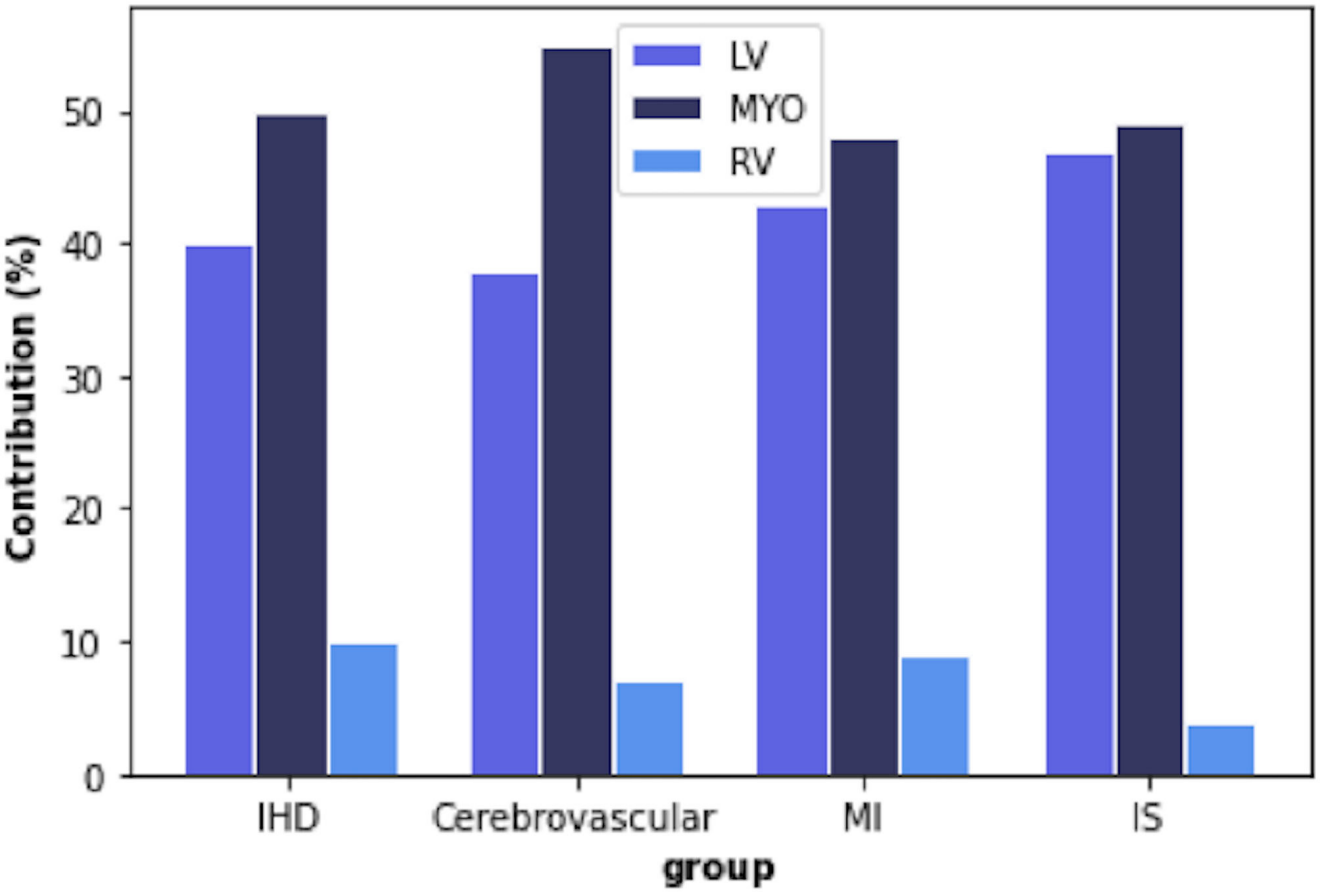
Percentage of radiomic features extracted from each region of interest (ROI) in each disease group. IHD, ischaemic heart disease; MI, myocardial infarction; IS, ischaemic stroke; LV, left ventricle; MYO, myocardium; RV, right ventricle.

Overall, the most informative features were those extracted from the LV blood pool region and the LV myocardium. In contrast, the RV cavity features had a minor role in discriminating the disease vs. healthy subgroups (Figure 4).

As the number of the most discriminative radiomics was high, we analysed the importance of each feature in the model, based on the mean weight value, to identify specific patterns for each disease group.

**Figure 5:**
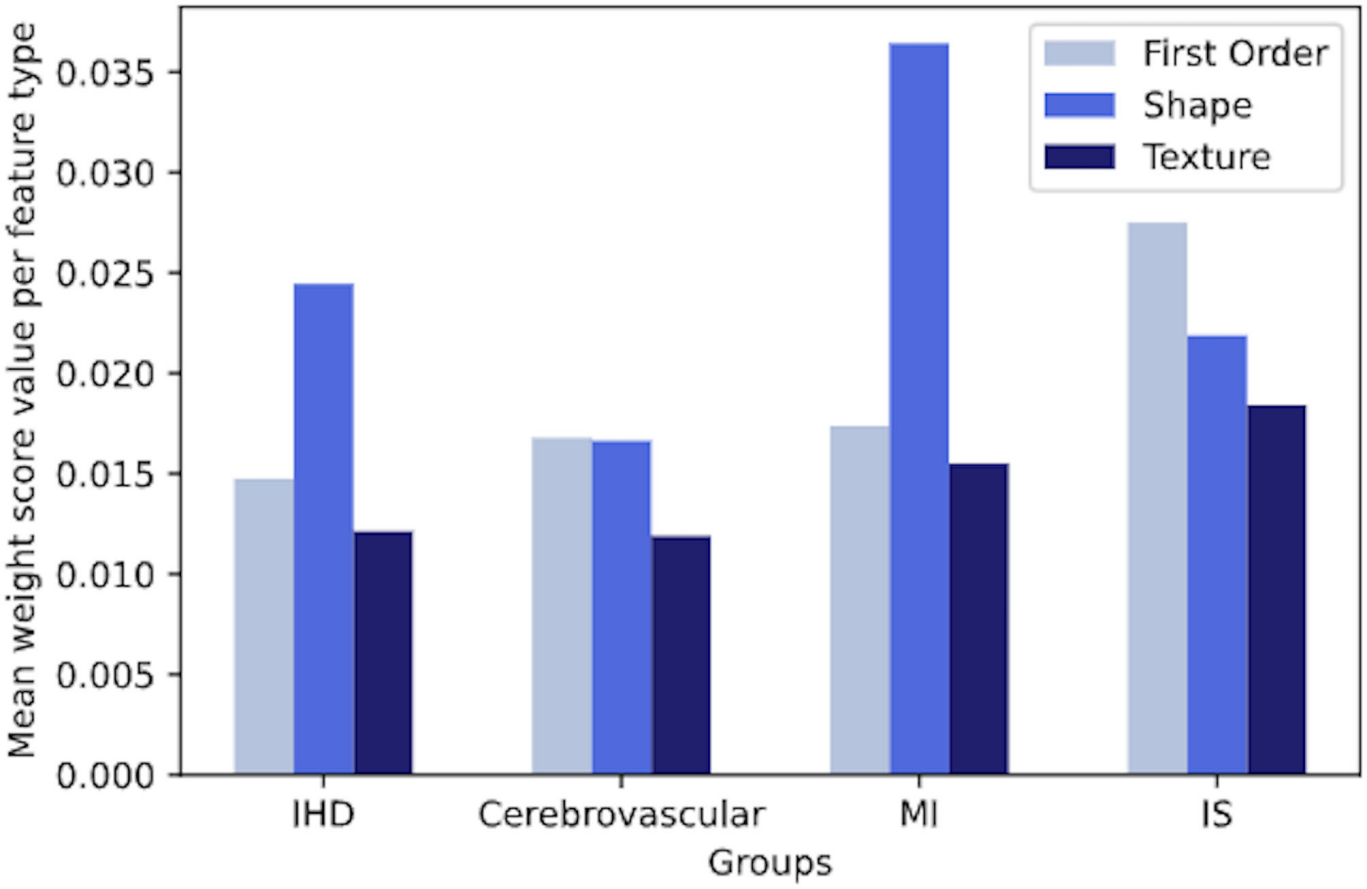
The overall contribution of first-order, shape, and texture features to a signature in IHD, cerebrovascular disease, MI and IS groups, based on the mean weight value per each feature type. IHD, ischaemic heart disease; MI, myocardial infarction; IS, ischaemic stroke.

We observed that both in IHD and MI, on average, shape features gave clearly more contribution per unit than intensity-based features. Instead, in cerebrovascular disease and IS, we did not observe a net dominance of one type of features over the other, although first-order and shape features were slightly more contributing to the signature (Figure 5).

This finding suggests that cardiac and cerebral ischaemic processes might impact the cardiac structures differently, and result in distinct cardiac imaging phenotypes on CMR radiomic analysis. Furthermore, there appears to be similar patterns of cardiac alterations between IHD and MI and between cerebrovascular disease and IS.

Such similarities were confirmed on further analysis of the radiomic features for each group. In this paper, we summarised only the results from the analysis of top-ranked features, for which we provided some possible clinically relevant interpretations.

### Comparing Radiomic Features Between IHD and MI

From the analysis of the top-ranked features, we found that shape radiomics were the most relevant in IHD and MI.

There were several features common to both conditions. For instance, the maximum 2D diameter of the LV and myocardium (slice/column/row), myocardial cavity volume, and surface area to volume ratio were highly featured in both diseases.

The direction of these features was also similar between the two conditions, reflecting common patterns of cardiac alterations. For instance, in both groups, there were larger cavity dimensions in end-diastole and end-systole compared to the healthy controls, as it was the volume of the LV myocardium at end-diastole. The mean value of the selected shape features for each disease group are shown in Table 3.

**Table 3:** Selected top-ranked most discriminative shape features for IHD and MI compared to healthy controls.

**Radiomic features**	**IHD**	**MI**	**Healthy controls**
Max 2D diameter slice (MYO) (ED)	75.767 ± 9.479	77.216 ± 8.121	72.632 ± 6.522
Max 2D diameter column (MYO) (ES)	87.168 ± 8.470	89.267 ± 8.776	83.676 ± 7.843
Surface area to volume ratio (MYO) (ED)	0.420 ± 0.055	0.413 ± 0.052	0.463 ± 0.059
Max 2D diameter column (MYO) (ED)	102.662 ± 8.627	104.478 ± 8.520	99.523 ± 8.307
Max 2D diameter slice (MYO) (ES)	66.201 ± 9.275	67.968 ± 8.713	62.503 ± 8.340
Max 2D diameter row (MYO) (ES)	86.460 ± 8.939	88.389 ± 8.990	83.389 ± 9.092
Volume (MYO) (ED)	91282 ± 22146	96595 ± 22534	78768 ± 20704
Least axis (MYO) (ES)	61.171 ± 6.379	63.351 ± 6.717	59.085 ± 5.528

*Data are presented as mean ± standard deviation. IHD, ischaemic heart disease; MI, myocardial infarction; MYO, myocardial; ED, end-diastole; ES, end-systole. All differences are statistically significant when compared with healthy controls (Bonferroni adjusted p < 0.05)*.

The prominence of these LV shape radiomics features indicates the tendency for the ischaemic cardiac disease to result in gross alterations of the LV geometry.

### Comparing Radiomic Features Between Cerebrovascular Disease and IS

Several shape, first-order, and texture radiomics were among the top-ranked features contributing to a radiomic signature in cerebrovascular diseases and IS.

Skewness, kurtosis, and sphericity of the LV were the most relevant features in both conditions. Furthermore, the direction of the changes described in comparison to healthy control was similar between the two groups.

For instance, the sphericity of the LV myocardium at end-diastole was greater amongst both disease groups compared with healthy individuals, indicating a less elliptical and more spherical chamber.

Furthermore, in both groups, there was less skewness in the distribution of signal intensity levels and a lower number of extreme intensities (kurtosis) within the LV blood pool region than the healthy controls.

The combination of all these findings indicate that ischaemic cerebrovascular diseases may be associated with subtle alterations both in the geometry and intra-cardiac haemodynamics of the LV.

Finally, texture radiomics played a significant role in improving disease discrimination in both cerebrovascular diseases and IS. Among the most informative texture radiomics, there were features describing the distribution of signal intensity values (e.g., low grey level run emphasis, large area emphasis), the textural heterogeneity (e.g., grey-level non uniformity, run-length non uniformity), and the relationship between voxel intensity and neighbourhood (e.g., large and small dependence high grey level emphasis). These features allowed a more granular tissue characterisation of the LV and myocardial tissue structure by quantifying the patterns of inter-voxel signal intensities. The analysis of texture radiomics revealed subtle differences at the tissue level between cerebrovascular disease and IS.

For instance, in cerebrovascular disease, there was less homogeneity among run lengths in the myocardial structure (run-length non-uniformity) as compared to the healthy comparators.

In IS, instead, there was lower variability of grey-level intensity values (grey-level non-uniformity) than healthy participants. These observations indicate that cerebrovascular disease may be associated with more textural heterogeneity of the LV myocardium than IS. These findings suggest that, despite some similarities, there may be some distinct alterations at the tissue level in the two conditions. The mean value of the selected radiomic features for each disease group are shown in Table 4.

**Table 4:** Selected top-ranked most discriminative shape, first-order and texture features for cerebrovascular disease and ischaemic stroke (IS) compared to healthy controls.

**Radiomic features**	**Disease group**	**Healthy controls**
**Cerebrovascular disease**		
Skewness LV ED (F)	−0.619 ± 0.170^*^	−0.742 ± 0.162
Sphericity MYO ED (S)	0.265 ± 0.021^*^	0.253 ± 0.017
Kurtosis LV ED (F)	2.390 ± 0.289^*^	2.610 ± 0.303
Run length non-uniformity	1,010.998 ± 214.912	957.296 ± 179.239
MYO ED (T)		
**Ischaemic stroke (IS)**		
Skewness LV ED (F)	−0.593 ± 0.165^*^	−0.742 ± 0.162
Sphericity MYO ED (S)	0.266 ± 0.020^*^	0.253 ± 0.017
Kurtosis LV ED (F)	2.355 ± 0.304^*^	2.610 ± 0.303
Grey level non-uniformity LV ES (T)	21.665 ± 6.258	22.472 ± 5.574

*Data are presented as mean ± standard deviation. S, shape radiomics; F, first-order radiomics; T, texture radiomics; LV, left ventricle; RV, right ventricle; MYO, myocardial; ED, end-diastole; ES, end-systole*.

* *Significant difference when compared with healthy controls (Bonferroni adjusted p < 0.05)*.

### Comparing Radiomic Signatures Between IHD and Cerebrovascular Disease

By referring to the constituent features within the radiomics signature for IHD and cerebrovascular disease, we were able to consider potential common biological pathways linking the two conditions (Table 5).

**Table 5:** Selected radiomic features for each disease group compared to healthy controls.

**Radiomic features**	**IHD**	**MI**	**Cerebrovascular** **disease**	**IS**	**Healthy**
Skewness LV (ED) (F)	−0.638 ± 0.173^*^	−0.649 ± 0.173^*^	−0.619 ± 0.170^*^	−0.593 ± 0.165^*^	−0.742 ± 0.162
Kurtosis LV (ED) (F)	2.435 ± 0.308^*^	2.452 ± 0.320^*^	2.390 ± 0.289^*^	2.355 ± 0.304^*^	2.610 ± 0.303
Sphericity MYO (ED) (S)	0.265 ± 0.021^*^	0.264 ± 0.021^*^	0.265 ± 0.021^*^	0.266 ± 0.020^*^	0.253 ± 0.017
Surface Area to volume ratio MYO (ES) (S)	0.279 ± 0.038^*^	0.281 ± 0.039^*^	0.279 ± 0.043^*^	0.279 ± 0.037^*^	0.298 ± 0.035
Surface Area to volume ratio MYO (ED) (S)	0.420 ± 0.055^*^	0.413 ± 0.052^*^	0.425 ± 0.059^*^	0.422 ± 0.053^*^	0.463 ± 0.059
Least axis MYO (ES) (S)	61.171 ± 6.379^*^	63.351 ± 6.717^*^^†^	60.041 ± 5.676	59.905 ± 5.544	59.085 ± 5.528

*Data are presented as mean ± standard deviation. IHD, ischaemic heart disease; MI, myocardial infarction; IS, ischaemic stroke; S, shape radiomics; F, first-order radiomics; LV, left ventricle; RV, right ventricle; MYO, myocardial; ED, end-diastole; ES, end-systole*.

* *Significant difference when compared with healthy controls (Bonferroni adjusted p < 0.05)*.

† *Significant difference when compared with the other disease groups (Bonferroni adjusted p < 0.05)*.

In general, radiomics shape features appeared more important for the signatures of the primary cardiac conditions (IHD and MI), indicating that gross anatomic alterations are important characteristics of these diseases. These features, instead, were less prominent within the cerebrovascular disease signatures.

There were also commonalities in the observed morphological alterations. For example, all disease categories considered showed greater sphericity and lower surface area to volume ratio of the LV myocardium compared to healthy cases, indicating a more spherical less elongated LV cavity shape.

Furthermore, MI and IHD showed significantly larger least axis of the LV myocardium at end-systole compared to controls, indicating a greater thickness of the myocardial wall in these conditions compared to healthy cases. Such changes were significantly greater in MI subjects compared to those with cerebrovascular diseases.

Thus, both IHD and cerebrovascular diseases had a more spherical LV shape. For IHD and MI, there was also significantly greater thickness of the LV myocardium and in general more prominence of shape alterations in the radiomics signature than with cerebrovascular disease.

For both IHD and cerebrovascular disease, intensity-features appeared important components of the radiomics signature, in particular skewness and kurtosis. All the disease categories considered had significantly less variation in signal intensity levels (skewness) within the LV blood pool region compared to healthy controls. Similarly, all the disease categories also showed significantly less Kurtosis (“pointiness”) of the LV blood pool intensities compared to controls. These prominent differences in signal intensity patterns within the LV blood pool may reflect common alterations in intra-cardiac haemodynamic associated with both IHD and cerebrovascular disease.

## Discussion

### Summary of Findings

This study demonstrates that CMR radiomics can capture changes in cardiac morphology, tissue or local structure in ischaemic heart disease and cerebrovascular disease even when ischaemia has not resulted in organ damage in form of MI or stroke.

Although conventional CMR indices identified some significant differences between the disease groups and the control, radiomics improved the quantification of alterations in both cardiac structure and tissue.

Radiomics only and combined models provided similar incremental value over conventional indices in discriminating MI and IS from healthy. Such value remained significant, albeit to a lesser extent, in identifying changes in more heterogeneous clinical pictures, such as IHD and ischaemic cerebrovascular diseases. This finding suggests that, although the clinical presentation of cardiac and cerebral ischaemia may vary, there may be common cardiac alterations detectable on radiomic analysis.

In particular, we observed that in cardiac ischaemia shape radiomics detected common changes in size and geometry of the LV and myocardium. In cerebral ischaemia, instead, the combination of shape, first-order and texture radiomics identified subtle alterations both in the geometry and tissue structure of the LV and myocardium, improving the disease discrimination significantly.

Furthermore, it appeared that there was a notable overlap of the radiomics signatures of IHD and cerebrovascular disease. This finding may reflect common risk factors, such as cardiometabolic morbidities, or alternative shared biological pathways implicated in both conditions.

The common cardiac alterations detected by radiomic features could represent the biological links between the brain and heart during the ischemic processes. Such alterations could also represent a valuable imaging marker for identifying individuals with previous cerebral ischaemia at risk of developing cardiac complications.

### Radiomic Analysis Improve Diagnosis of Cardiac and Cerebral Ischaemia

Our study confirms the capacity of CMR radiomics to improve the accuracy of diagnosing important diseases over conventional image analysis.

Previous studies have demonstrated the ability of radiomics to accurately diagnose myocardial infarction on non-enhanced images. For instance, Baessler et al. (26) showed that five independent texture radiomics allowed to differentiate ischaemic scar from normal myocardium on cine CMR images. Furthermore, Larroza et al. demonstrated that texture analysis could discriminate acute MI from chronic MI both on contrast and cine CMR images, where MI is often visually imperceptible. In particular, the combinantion of 75 texture features provided high disease discrimination on cine CMR images (27).

Similarly, we found that radiomic analysis of cine CMR images allowed to discriminate MI from healthy comparators with significantly superior performance to conventional CMR indices.

In contrast to the previous studies, in this work we analysed all three types of radiomic features. We found that in MI, shape radiomics were the most important feature in the classification models. Similar findings were observed in IHD group, which included a large spectrum of clinical conditions, that vary from stable angina to acute coronary syndrome (ACS).

The analysis of shape features revealed similar changes in size, particularly in the long axis, and in the geometry of the LV myocardium, between IHD and MI.These findings might indicate that cardiac ischaemia, whether or not it results in organ damage, might lead to typical cardiac remodelling patterns that involve the LV globally.

In all the clinical scenarios characterising IHD, the myocardial energy imbalance plays a central role in leading to ischaemia (28). We hypothesise that the alterations found in IHD may be due to a chronic reduction of the coronary flow reserve, which could lead to a lower supply of oxygen to the myocardium and, over the time, to LV myocardial remodelling (29).

Therefore, the radiomic signature for IHD may represent an additional non-invasive imaging marker of cardiac ischaemia. It might improve the diagnosis of a wide variety of conditions, including angina pectoris and silent ischaemia, without using late gadolinium enhancement (LGE) tecnique.

Our study confirms also the potential of radiomics to identify cardiac changes associated with important conditions that do not affect the heart exclusively.

We found that radiomic-based models provided high degrees of discrimination of ischaemic cerebrovascular disease and IS. In particular, we observed that the combination of shape, first-order and texture radiomics in the classification models led to the highest accuracy of diagnosis. Perhaps, intensity-based radiomics allowed more granular disctinction of cerebral ischaemia enhancing further the diagnostic value of the classification models.

Similarly, Cetin et al. (30) found that first-order and texture radiomics significantly improved detection of early effect of certain cardiovascular risk factors on cardiac structure and tissue, such as diabetes and smoking.

In particular, the median intensity of the myocardium and grey level non-uniformity were described as the single most discriminative radiomic to identify diabetes and current smokers, respectively.The grey level non-uniformity has been also described as the single most important feature in identifying hypertrophic cardiomyopathy (HCM) (31). Interestingly, we found that this textural radiomic feature was among the most discriminative features in cerebral ischaemia, particularly in IS. Probably such similarities might be due to common patterns of tissue fibrosis associated with those particular conditions that are reflected in the observed texture features.

Finally, it should be noted that although some features were more representative than others, we observed that their combination significantly improved the diagnostic accuracy in each disease group. We speculate that the clinical and pathophysiological heterogeneity of the conditions examined might translate into a complex variety of subtle cardiac changes reflected in the observed features. Therefore, it was the combination of all these alterations that made a distinct signature for cardiac and cerebral ischaemia.

### CMR Radiomics Analysis to Improve Understanding of the Brain-Heart Interactions

CMR radiomics analysis uncovered common patterns of cardiac alterations in ischaemic heart and cerebrovascular diseases, which might represent the imaging markers of the biological interactions between brain and heart during the ischaemic processes.

Such similarities might reflect shared cardiovascular risk factors involved in the common brain-heart disease crosstalk, probably mediated by atherosclerosis and arteriosclerosis (32).

Furthermore, there is growing evidence suggesting that subjects who suffered cerebral ischaemic events might be highly vulnerable to cardiac complications, even in the absence of risk factors or pre-existing heart disease (5, 33). Although most of the cardiac alterations tend to resolve entirely over the following weeks from an acute event, some of them can persist, causing poor early and long-term outcomes and death (8–10, 34).

This suggests that alternative mechanisms of brain-heart interactions, not mediated by shared risk factors, might be involved during the ischæmic processes. For instance, high circulating catecholamine level, sympathetic/parasympathetic unbalance, and stroke-related systemic inflammation mediated by cytokines, have been described as possible causes of brain damage-induced cardiac dysfunction. Other mechanisms involved in the associations include dysfunction of the hypothalamic-pituitary-adrenal axis, brain blood barrier damage, and gut microbiome dysbiosis (33, 35).

Previous studies have suggested that endothelial inflammation, oxidative stress, and catecholamine release induced by ischaemic brain injury may lead to chronic myocardial dysfunction and remodelling, accelerating atherosclerosis, and vasoconstriction of the coronary arteries (35). Furthermore, there is growing evidence that even subclinical cardiac dysfunctions, such as diastolic dysfunction, might be associated with clinical stroke or silent infarcts on magnetic resonance imaging (MRI) scans, especially in older adults (36).

Whether such cardiac dysfunctions are triggered by the ischaemic brain damage, are unrelated complications, or just the underlying causes is still unclear and require further investigations. Nevertheless, independently of the pathogenesis, these associated cardiac abnormalities may become the substrate of future cardiovascular events, such as heart failure or CAD, and further affect patients' outcome (37, 38).

Cardiac imaging represents a valuable diagnostic tool for identifying the aetiology of cerebrovascular disease and detecting cardiac comorbidities. The main long-term cardiac abnormalities described on conventional images are the LV systolic and diastolic dysfunction, which in conjunction with the increased burden of arrhythmia, lead to cardiac remodelling (8).

We found that cerebral ischaemia was associated mainly with changes in the geometry and tissue structure of the LV and myocardium, the latter affecting the cardiac texture appearance in CMR images. In particular, we found that individuals with ischaemic cerebrovascular disease had less heterogeneous myocardial tissue textures than healthy comparators. We may speculate that these features represent different levels of myocardial damage in these patients. Perhaps, the toxic effect of catecholamines on the myocardium and the subsequent neurogenic hypertension are two possible mechanisms that may lead to chronic myocardial dysfunction and remodelling (39).

Such subtle cardiac alterations, especially at the tissue level, often remain undetected to traditional CMR metrics or echocardiography. In our study, CMR radiomics provided deeper image phenotyping than conventional indices improving detection of changes in cardiac structure and tissue associated with cerebral ischaemia, thus making the diagnosis more precise.

Radiomics analysis might improve our understanding of cerebral ischemia's early effects on the cardiac structure and tissues. Identifying imaging markers of adverse brain-heart disease at an early stage may enable improved clinical management and reduce mortality in patients with previous cerebral ischaemia. These markers may represent a potential intervention target to mutually protect the heart after stroke and the brain from further damage. An integrated approach based on appropriate risk stratification and early screening for cardiac dysfunction, coupled with aggressive risk factor control and early treatment, is essential in managing patients with an ischemic event.

### Study Limitations and Future Directions

To our knowledge, this is the first study using CMR radiomics to study the cardiac changes associated with cardiac and cerebral ischaemia. We used clinical and imaging information from the UKB dataset, which has extensive data available from each participant, making it an excellent resource to address brain-heart interactions. We used a combination of ICD-10 and ICD-9 codes to select our study populations to avoid concerns about the self-reported conditions' accuracy and objectivity. However, the estimated prevalence and diagnostic accuracy of certain conditions, such as angina or TIA, may be affected by the hospital information system and the quality of the documentation recorded.

Furthermore, the identification of participants with MI was only based on the clinical diagnosis. However, further information on the myocardial scar extent was not available in the UKB dataset. Therefore, in this study we could not determine whether there was a correlation between the radiomic features and the myocardial scarring extent.

There were fewer subjects with cerebrovascular disease and IS than those with IHD and MI. Therefore, despite the significant discriminatory power of radiomics-based models observed in cerebral ischaemic conditions, these results must be interpreted cautiously.

Radiomics are highly dependent on image acquisition factors that can greatly modify texture and histogram-based intensity values. Different centres' datasets should be evaluated to confirm validity of these machine learning algorithms in a large-scale application.

Furthermore, the number of radiomics needed to achieve better performances can be considered large. Despite the automatic and fast procedure to perform this test in a real clinical scenario, reduction of these subsets must be achieved in the future, to build more interpretable and explainable tools.

We did not match the healthy comparators and the disease groups on a per-patient basis. Therefore, we must acknowledge that the higher proportion of females and the younger age of the healthy controls might have partially influenced the radiomic values and thus the disease prediction. However, these limitations were mitigated by the fact that the control subjects, according to the robust definition of “health” proposed by Petersen et al. (18), did not have any risk factors or conditions that might have affected the heart more significantly.

Finally, we did not study the correlation between radiomics and patients' risk factors and time from the ischaemic event, as that was beyond this paper's purpose. More extensive prospective studies using external validation cohorts and accounting for confounding factors are needed to determine these models' clinical utility.

## Conclusions

This study demonstrates the potential value of CMR radiomics over conventional indices in detecting subtle cardiac changes associated with chronic ischaemic processes involving the brain and heart, even in the presence of more heterogeneous clinical pictures. Radiomics analysis might improve our understanding of the effects of cerebral ischaemia on cardiac structure and tissue contributing to shed light on the complex brain-heart interactions.

## Data Availability Statement

The datasets presented in this study can be found in online repositories. The names of the repository/repositories and accession number(s) can be found below: http://www.ukbiobank.ac.uk/register-apply/.

## Ethics Statement

This study was reviewed and approved by the NHS National Research Ethics Service (17th June 2011, Ref11/NW/0382) and extended on 10th May 2016 (Ref16/NW/0274). Written informed consent was provided by participants. The patients/participants provided their written informed consent to participate in this study.

## Author Contributions

ER, SP, and KL conceived the idea and designed the study. ZR-E provided support on the clinical aspect of the study. PG and CI performed the radiomics analysis and designated the machine learning methods. KL supervised radiomics analysis. NA provided cardiac measures from automated analysis pipeline. ER wrote the manuscript. CI drafted the part relative to the machine learning and radiomic analysis. All authors contributed to the articles, read, and provided critical revision of the manuscript.

## Funding

ZR-E is supported by a British Heart Foundation Clinical Research Training Fellowship (FS/17/81/33318). SP acknowledges support from the National Institute for Health Research (NIHR) Biomedical Research Centre at Barts. SP acknowledge the British Heart Foundation for funding the manual analysis to create a cardiovascular magnetic resonance imaging reference standard for the UK Biobank imaging resource in 5,000 CMR scans (www.bhf.org.uk; PG/14/89/31194). SP acknowledges support from the SmartHeart EPSRC programme grant (www.nihr.ac.uk; EP/P001009/1). SP and ER also acknowledge support by the London Medical Imaging and Artificial Intelligence Centre for Value Based Healthcare (AI4VBH), which is funded from the Data to Early Diagnosis and Precision Medicine strand of the government's Industrial Strategy Challenge Fund, managed and delivered by Innovate UK on behalf of UK Research and Innovation (UKRI). NA was supported by a Wellcome Trust Research Training Fellowship (wellcome.ac.uk; 203553/Z/Z). NA recognises the National Institute for Health Research (NIHR) Integrated Academic Training programme which supports his Academic Clinical Lectureship post. This work was partly funded by the European Union's Horizon 2020 research and innovation programme under grant agreement no. 825903 (euCanSHare project). KL received funding from the Spanish Ministry of Science, Innovation and Universities under grant agreement RTI2018-099898-B-I00.

## Author Disclaimer

The views expressed are those of the authors and not necessarily those of the AI4VBH Consortium members, the NHS, Innovate UK, or UKRI.

## Conflict of Interest

SP provides consultancy to and owns stock of Cardiovascular Imaging Inc, Calgary, Alberta, Canada. The remaining authors declare that the research was conducted in the absence of any commercial or financial relationships that could be construed as a potential conflict of interest.

## Publisher's Note

All claims expressed in this article are solely those of the authors and do not necessarily represent those of their affiliated organizations, or those of the publisher, the editors and the reviewers. Any product that may be evaluated in this article, or claim that may be made by its manufacturer, is not guaranteed or endorsed by the publisher.

## Abbreviations

ACS: Acute coronary syndrome
AUC: Area under the ROC curve
BSA: Body surface area
CMR: Cardiovascular magnetic resonance
ED: End-diastole
ES: End-systole
FS: Feature selection
HCM: Hypertrophic cardiomyopathy
HLA: Horizontal long-axis
IHD: Ischaemic heart disease
IS: Ischaemic stroke
LAX: Long-axis
LGE: Late gadolinium enhancement
LV: Left ventricle
LVEDV: Left ventricle end-diastolic volume
LVEF: Left ventricle ejection fraction
LVESV: Left ventricle end-systolic volume
LVM: Left ventricle mass
LVOT: Left ventricular outflow tract
LVSV: Left ventricle stroke volume
MI: Myocardial infarction
ML: Machine learning
MRI: Magnetic resonance imaging
MYO: Left ventricle myocardium
RF: Random forest
ROC: Receiver operating characteristic
ROI: Region of interest
RV: Right ventricle
RVEDV: Right ventricle end-diastolic volume
RVEF: Right ventricle ejection fraction
RVESV: Right ventricle end-systolic volume
RVSV: Right ventricle stroke volume
SAX: Short-axis
SI: signal intensity
SFS: Sequential feature selection
SVM: Support vector machines
TIA: Transient ischamic attack
UKB: United Kingdom Biobank
VLA: Vertical long-axis.
