# Supplementary material for: New Imaging Signatures of Cardiac Alterations in Ischaemic Heart Disease and Cerebrovascular Disease Using CMR Radiomics
Source: Front Cardiovasc Med. 2021 Sep 23;8:716577. doi: 10.3389/fcvm.2021.716577 (PMC8494975; doi:10.3389/fcvm.2021.716577)

Supplementary Figure 1. Receiver operating curves (ROC) for the best performing combined models in each disease group (Panel A-D). SVM: Support Vector Machines; IHD: ischaemic heart disease; MI: myocardial infarction; IS: ischaemic stroke.

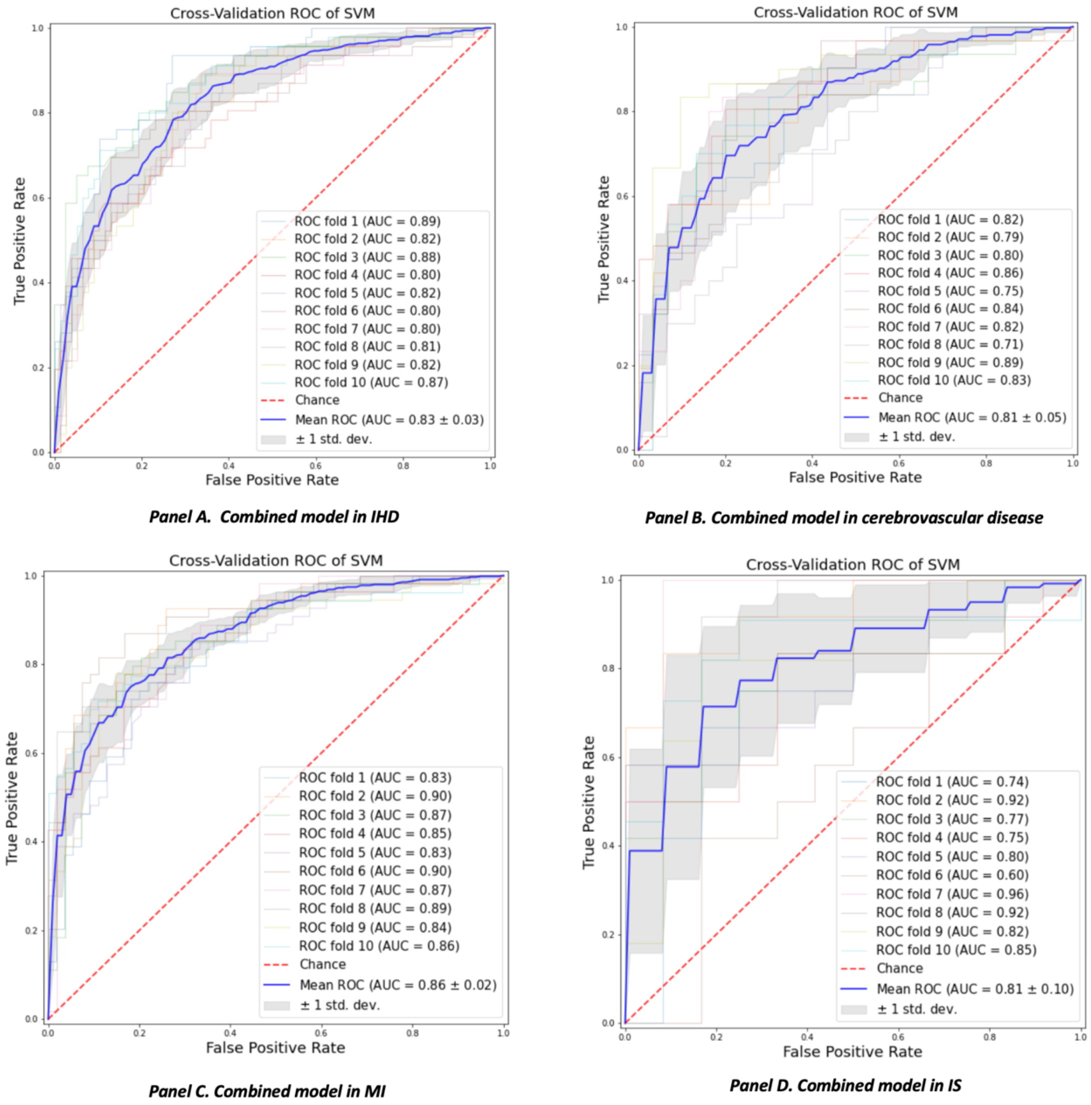

Supplement: Supplementary file 1 [file Image_1.PDF]
