# Supplementary material for: New Imaging Signatures of Cardiac Alterations in Ischaemic Heart Disease and Cerebrovascular Disease Using CMR Radiomics
Source: Front Cardiovasc Med. 2021 Sep 23;8:716577. doi: 10.3389/fcvm.2021.716577 (PMC8494975; doi:10.3389/fcvm.2021.716577)
Supplement: Supplementary file 2 [file Table_1.PDF]

Supplementary Table 1. List of ICD10 (field 41270) and ICD9 (field 41271) codes used to define the ischaemic heart disease (IHD), myocardial infarction (MI), cerebrovascular disease, and ischaemic stroke (IS) cohorts from the UK Biobank datasets.

**Ischaemic heart disease (IHD)**	
ICD10	I20, I21, I22, I23, I24, I25
ICD9	410, 411, 412, 413, 414
**Myocardial infarction (MI)**	
ICD10	I21, I22, I23, 124.1; 125.2
ICD9	410; 411; 412
**Cerebrovascular disease**	
ICD10	I63, I64, G45
ICD9	433, 434, 435, 436
**Ischaemic stroke (IS)**	
ICD10	I63
ICD9	433, 434
