# Supplementary material for: New Imaging Signatures of Cardiac Alterations in Ischaemic Heart Disease and Cerebrovascular Disease Using CMR Radiomics
Source: Front Cardiovasc Med. 2021 Sep 23;8:716577. doi: 10.3389/fcvm.2021.716577 (PMC8494975; doi:10.3389/fcvm.2021.716577)
Supplement: Supplementary file 3 [file Presentation_1.PDF]

More extensive description of the Machine learning models building

Our model is based on three different points: dimensionality reduction, machine learning (ML) modelling and results interpretation.
Radiomics were extracted following the default pipeline from Pyradiomics (1). A total of 446 radiomics remained after removing the RV intensity-based features. A high correlation among them was assumed. Therefore, we applied a highly correlated removal procedure using Pearson correlation > 0.9 to remove redundant features. This algorithm selected a feature from the beginning of the dataset and compared it with the rest of the features sequentially one by one. The initial radiomics extraction was performed, so that shape features were placed in the first positions of the data frame, followed by the first-order and texture features. In this way, shape features (which are much easier for interpretation) were selected over intensity-based features in case of high correlation. Thus, this method allowed enhancing results explainability.

The highly correlated removal procedure led to a final number of approximately 200 (± 12) radiomic features for each study group. Next, the reduced data subset was introduced in a scikit-learn Pipeline that included a scaling MinMaxScaler function from Python's scikit-learn library (2) and feature selection (FS) algorithm called KBest (2). This FS technique was followed by the selected ML model, altogether combined in a Grid Search for hyper-parameter tuning optimization (Table 1).

The models were validated following a nested 10-fold K Stratified cross validation criteria for the outer loop (Outer CV), and a 3KFold for the inner loop (Inner CV), always respecting balance for the classes (Figure 1).

Several ML algorithms were tested, but only two were selected among them: Support Vector Machine (SVM), as the top performer, and Random Forest (RF), the second top performer and one of the most interpretable ML algorithms. We performed a paired t-test across folds to evaluate the statistical significance in the comparison and the p-values obtained for each model. Receiver operating characteristic (ROC) curves were calculated, for conventional CMR indices and radiomics and the combination of both (combined model). To avoid variations when determining the most relevant features, we analysed the validation area under the curve (AUC) score value for each subset reduction and averaged across folds and for all hyper-parameters. Once features were selected and their respective weights were provided, we obtained the average weight per fold. Finally, we sorted the most relevant features according to the previously obtained subset reduction number.

For the 3rd model experiment that combined radiomics and conventional CMR indices, we evaluated the correlation from CMR indices first, ensuring to keep all of them and removing only the redundant radiomics. This was done to ensure that we were testing the added value of radiomics.

Table 1. Hyperparameters tuned in our Grid search for SVM and RF models. SVM: Support Vector Machines; RF: Random Forest.

Model	Hyperparameter tuned	Values
SVM	C	[0.01,1,10,100,1000]
	gamma	[1,0.1,0.001,0.0001]
	Kernel	Linear, RBF
RF	Bootstrap	True, False
	Min_samples_leaf	[3, 4, 5, 6]
	Min_samples_split	[8, 10, 12]
	N_estimators	[100, 500, 1000]

Figure 1. A descriptive pipeline template for the nested cross-validation method. Variations in the number of splits for the outer loop (Outer CV) and Inner loop (Inner CV) can change according to the experimentation or number of samples.

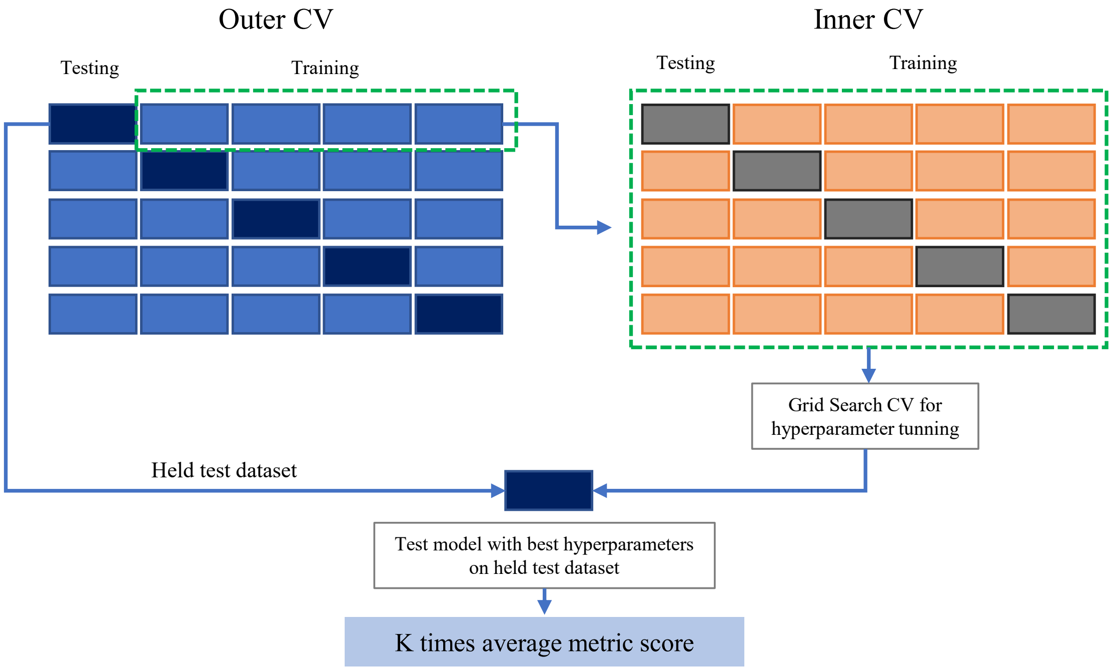

REFERENCES

1. van Griethuysen JJM, Fedorov A, Parmar C, Hosny A, Aucoin N, Narayan V, et al. Computational Radiomics System to Decode the Radiographic Phenotype. *CancerRes* (2017). 77: e104-e107. doi: 10.1158/0008-5472.CAN-17-0339.
2. Pedregosa F, Varoquaux G, Gramfort A, Michel V, Thirion B, Grisel O, *et al*. (2011). Scikit-learn: Machine Learning in Python. *JMLR.* 12: 2825-2830. *Available at: http://jmlr.org/papers/v12/pedregosa11a.html.*
